# Impact of differential detection of TM6SF2 rs58542926 mutation in circulating tumor DNA versus peripheral blood cells on hepatocellular carcinoma patients

**DOI:** 10.1007/s12672-025-02812-9

**Published:** 2025-06-12

**Authors:** Samar Samir Youssef, Eman Abd El Razek Abbas, Mohamed Hassany, Aya Shaaban Ragheb Shaaban, Eman Elsayed Abass Mohammed, Tamer Elbaz

**Affiliations:** 1 Microbial Biotechnology Department, Biotechnology Research Institute, National Research Centre, Dokki, Egypt; 2 Tropical Medicine Department, National Hepatology and Tropical Medicine Research Institute, Cairo, Egypt; 3 Biochemistry Department, Faculty of Science, Ain Shams University, Cairo, Egypt; 4 Medical Molecular Genetics Department, Human Genetics and Genome Research Institute, National Research Centre, Dokki, Egypt; 5 Endemic Hepatology and Gastroenterology, Faculty of Medicine, Cairo University, Cairo, 11562 Egypt; 6 Newgiza University, Faculty of Medicine, Giza, Egypt

**Keywords:** Hepatocellular carcinoma, HCC, TM6SF2 mutation, HCV infection

## Abstract

This study aimed to investigate the differential distribution of the genotypes of the TM6SF2 rs58542926 polymorphism in circulating tumor DNA (ctDNA) versus peripheral blood cells (PBMC) and its association with the development of hepatocellular carcinoma (HCC) in patients infected with the hepatitis C virus (HCV) in Egypt. A total of 147 HCC patients were enrolled in the study, and their genomic and ctDNA were analyzed for the TM6SF2 rs58542926 genotype. The CC genotype was identified as the most common among patients with HCC in Egypt, while the CT genotype was associated with higher serum direct bilirubin, total leucocyte count, and hemoglobin levels than those with the CC genotype. Circulating samples from patients with the CT genotype had significantly higher serum direct bilirubin, ALT, and prothrombin time than those with the CC genotype, as well as a significantly higher percentage of smokers, alcohol intake, and HCV PCR-positive patients. Our findings suggest that lifestyle factors such as smoking and alcohol intake may contribute to the development of HCC in patients with the CT genotype. Both genomic and ctDNA for the TM6SF2 rs58542926 genotype complemented and correlated with parameters of chronic liver disease and underlying risk factors for HCC. Circulating DNA for gene polymorphism provided additional real-time information and more value in correlation with the worsening of liver condition and the presence of HCC risk factors.

**Supplementary Information:**

The online version contains supplementary material available at 10.1007/s12672-025-02812-9.

## Key messages

- Our study provides evidence for the differential distribution of genotypes of TM6SF2 rs58542926 gene polymorphism in PBMC versus ctDNA and both correlate with the development of HCC in patients with HCV infection.
- Alteration in TM6SF2 rs58542926 genotypes in ctDNA correlated with worsening of liver condition indicating its impact on disease progression monitoring.
- Our findings suggest that smoking and alcohol intake may contribute to the development of HCC in patients with the CT genotype of TM6SF2 rs58542926.
- Our results may help to inform future research and clinical practice in the diagnosis and treatment of HCC in patients with HCV infection.

**Supplementary Information:**

The online version contains supplementary material available at 10.1007/s12672-025-02812-9.

## Introduction

Globally, HCC is the sixth most common cause of cancer-related deaths and the third-leading factor in cancer-related years of life lost [1]. Asia and Africa have the greatest global incidence rates. In Egypt, HCC accounts for 33.63% of all cancers in males and 13.54% in women, it is considered a severe public health issue [2]. Among the various causes of HCC in Egypt, HBV and HCV infection are the predominant causes [3]. Worse prognosis is linked to several demographic (such as male gender), clinical (such as lymphatic vessel density) and labortaory characteristics (such as alpha fetoprotein and higher ALBI grades) regardless of line of treatment applied [4, 5]. Furthermore, it has been established that genetic mutations impact a person’s vulnerability to liver cancer. The pathophysiology of liver cancer is linked to genetic factors, which also increase individual differences in disease susceptibility [6].

Through genome-wide association studies (GWAS), single-nucleotide polymorphisms (SNPs) have been found in different genes, including the TM6SF2 gene, and have been linked to the spectrum of non-alcoholic fatty liver disease (NAFLD) and so HCC [7–9]. Moreover, a study by Youssef et al*.* in 2022 suggests a probable relationship between HCC development occurrence and TM6SF2 variants in HCV-related HCC patients in Egypt [10]. The TM6SF2 gene is found on chromosome 19 and is involved in lipid metabolism [11]. The rs58542926 C > T variant in the TM6SF2 gene shows a glutamate substitution with lysine at the 167 (E167 K) locus that raises the level of intrahepatic TAG and induces the liver to retain very-low-density lipoprotein (VLDL) [11].

Hepatic biopsy remains the gold standard for yielding clinically significant information regarding a patient’s diagnosis, prognosis, or management, and so for research purposes. One of the most significant goals of hepatological research in recent years has been the discovery of non-invasive biomarkers in order to mimic complication challenges associated with sample extraction [12].

Liquid biopsy is a term used to describe the molecular examination of tumor molecules extracted from solid tumors and transferred into biological fluids such as blood. Circulating tumor DNA (ctDNA) is one of these circulating analytes. Recently, tissue-specific abnormalities can be fortunately identified by circulating tumor DNA (ctDNA), which has drawn great interest as a potential tool for prognosis, diagnosis, and therapy follow-up, demonstrating promising outcomes in the cancer field, including HC [13–15]. While ctDNA refers to the percentage of tumor DNA in the cfDNA pool, cfDNA is defined as cell-free DNA extracted from blood, which comprises DNA released from all cells, including normal cells and cells participating in pathologic processes (such as inflammation and neoplasia). Tumor cells release circulating tumor DNA (ctDNA) into the bloodstream, which contains the original tumor’s mutations including point mutations, chromosomal rearrangements, and copy number variations [16]. Next-generation Sequencing (NGS) is a very powerful technology for nucleic acid sequencing and detection of mutations accurately even on the whole genome sequence thus, it is used in a wide range for diagnosis, may be at early stages, detection of treatment methods and follow up of patients [17]. An early study predicted that NGS has the potential to uncover somatic genetic changes within a cancer genome by sequencing DNA from both the tumor and the germline [18]. According to a different study, next-generation technology for cell-free DNA (cfDNA) sequencing may be used to monitor and select therapies for patients with metastatic breast cancer (MBC), and they already showed a promising result by using machine learning in clearing somatic variants depending on cfDNA sequencing [19].

This study aims to determine the difference in genetic distribution between cfDNA and genomic DNA from peripheral blood mononuclear cells (PBMCs) concerning the TM6SF2 rs58542926 polymorphism in HCC patients.

## Subjects and methods

### Study population

Patients with HCC were enrolled at the National Hepatology and Tropical Medicine Research Institute, in Cairo. HCC patients were diagnosed according to the European Association for the Study of the Liver (EASL) guidelines by ultrasonography and multiphasic computed tomography (CT). All patients had been previously infected with hepatitis C virus. Baseline clinical data were collected from all participants, which included serum alpha-fetoprotein (AFP), liver function, tumor size and site, portal vein thrombosis (PVT), ascites and the stage of HCC classified by the Barcelona Clinic Liver Cancer (BCLC) staging system. Only HCC patients at stages 0, A, and C were included in the study.

A total of 147 participants were enrolled in the study, including 72 patients with HCC in the early stage (BCLC stage 0 and A) and 75 patients with HCC in the late stage (BCLC stage C). The exclusion criteria for all HCC patients included HBV or co-infection with other viruses, HIV infection or liver metastasis from other cancer types. All patients signed an informed consent and the study was conducted according to the ethical principles of Declaration of Helsinki. This study was approved by the Ethics Committee of the Medical Research, registration number (022052023), at National Research Centre, in Dokki, Cairo, Egypt.

### Blood sampling and DNA extraction

Ten milliliters of blood were collected in EDTA tubes from patients with HCV-related HCC. Plasma and the buffy coat layer were isolated from blood samples from each patient to compare them with each other. Plasma was purified by 2 steps of centrifugation, the first step was at 1900 xg for 10 min at room temperature (15–25 ℃) to separate plasma from other blood components without disturbing the buffy coat layer and also separate the buffy coat, and the second step was at 16,000 × g for 10 min at 4 ℃ to discard debris from plasma without disturbing the pellet, and the plasma was stored at −80°c until use. The buffy coat layer which was isolated from the same patients was stored at 20 ℃.

The cfDNA was extracted from 4 mL plasma using the QIAamp® Circulating Nucleic Acid (Qiagen#55114), following the manufacturer’s instructions and preserved at –20 °C for genetic determinations. Germline DNA (genomic DNA) was extracted from the buffy coat layer using QIAamp DNA Blood Mini kit (Qiagen #51104) according to the manufacturer’s instructions and preserved at –80 °C for genetic determinations. The Thermo Scientific NanoDrop 2000c (Thermo Fisher Scientific, Invitrogen) was used to quantify the purified cfDNA and gDNA. The quality of DNA was assessed by 1.5% agarose gel electrophoresis.

### Genotyping TMS6 F2 rs58542926 polymorphism

After cfDNA and gDNA extraction, real-time PCR was used to analyze the polymorphism of the TMS6 F2 rs58542926 using the “Taqman allelic discrimination assay” on Agilent Mx3000p qPCR, (Agilent Technologies, Germany) as described previously (10). The interpretation of the TMS6 F2 rs58542926 genotype was given by (CC, CT, TT).

### Statistical analysis

The sample size was calculated by using (PASS 11, NCSS, LLC, Kaysville, Utah, USA) software. Data were analyzed using IBM SPSS advanced statistics (Statistical Package for Social Sciences), version 24 (SPSS Inc., Chicago, IL). Numerical data were described as mean and standard deviation, median, and range. Data were explored for normality using the Kolmogorov–Smirnov test and the Shapiro–Wilk test. Comparisons between 2 groups for normally distributed numeric variables were done using the independent t-test, while for non-normally distributed numeric variables, they were done using the Mann–Whitney test. Categorical data were described as numbers and percentages, and comparisons were done by the chi-square test or Fisher exact as appropriate. Logistic regression was used to estimate factors affecting depression; all significant variables in univariate analysis were entered into the model. A p-value less than or equal to 0.05 was considered statistically significant. All tests were two-tailed.

## Results

This study comprised 147 Egyptian patients (n = 30 females and n = 117 males) with HCC at different stages. It included both the circulating free DNA and the genomic DNA of the patients from the same sample. The distribution of the TM6SF2 rs58542926 genotypes was examined on the two DNA types.

### Correlation between genotyping of TM6SF2 rs58542926 concerning two different DNA types

Based on the genotyping results of 147 HCC patients, 132 patients had a CC genotype and 15 had a CT genotype in the circulating DNA, while 135 patients had a CC genotype and 12 had a CT genotype in the genomic DNA (Table 1). This highlights the fact that 15 samples had their genotypes changed from CC to CT and from CT to CC, respectively.

**Table 1:** Different TM6SF2 genotypes involved in genomic and circulating DNA

Sample Type Genotype	Count	%
Genomic DNA		
CC	135	91.8%
CT	12	8.2%
Circulating DNA		
CC	132	89.8%
CT	15	10.2%

### Correlation between patients’ laboratory investigations and different TM6SF2 rs58542926 genotypes involved in genomic and circulating DNA

Table 2 displays the relationship between TM6SF2 rs58542926 genotypes and laboratory results for 147 HCC patients. Significantly higher levels of hemoglobin, serum direct bilirubin, and total leucocytic count were found in patients with the CT genotype (n = 12) in their genomic DNA compared to those with the CC genotype (n = 135). However, patients with the CT genotype in their circulating DNA (n = 15) exhibited noticeably higher levels of ALT, serum direct bilirubin, prothrombin time, and INR than those with the CC genotype (n = 132).

**Table 2:** Baseline laboratory investigations and ALU ratio of patients concerning different TM6SF2 genotypes in genomic and circulating DNA

	Genomic DNA											Circulating DNA						
	CC	n = (135)				CT		(n = 12)			*P* value	CC (n = 132)			CT (n = 15)			*P* value
	Median	1 st quartile		3rd quartile		Median		1 st quartile		3rd quartile		Median	1 st quartile	2nd quartile	Median	1 st quartile	2nd quartile	
ALU ratio	1.34	1.30		1.36		1.37		1.31		1.46	0.066	1.34	1.30	1.36	1.35	1.28	1.38	0.735
WBCs (x* 10^3^)	4.70	3.90		6.20		7.95		6.45		8.65	< 0.001	4.75	4.00	6.25	5.40	4.10	8.40	0.313
Hb (mg/dL)	12.40	11.90		13.60		13.35		12.75		14.50	0.013	12.40	12.05	13.65	11.60	11.50	13.60	0.261
Platelets (x/mm^3^)	121.00	96.00		158.00		205.50		122.50		233.00	0.056	125.00	100.50	183.00	75.00	66.00	163.00	0.074
AST (U/L)	43.00	35.00		77.00		59.50		43.00		103.00	0.143	44.00	34.50	76.00	64.00	37.00	86.00	0.195
ALT (U/L)	39.00	32.00		59.00		50.00		36.00		67.50	0.214	38.50	31.00	56.00	60.00	59.00	67.00	0.003
Creatinine (mg/dL)	0.98	0.87		1.10		1.16		0.85		1.33	0.117	0.98	0.88	1.12	1.00	0.70	1.35	0.643
AFP (μg/L)	10.92	5.30		49.60		14.18		3.52		29.30	0.408	10.22	5.15	39.30	11.70	8.90	35.30	0.195
Albumin (g/dL)	3.60	3.03		3.87		3.73		2.85		4.08	0.873	3.60	3.01	3.89	3.30	3.10	3.95	0.818
Total bilirubin (mg/dL)	1.00	0.80		1.32		1.28		1.03		1.60	0.118	1.00	0.75	1.31	1.26	0.90	1.90	0.061
Direct bilirubin (mg/dL)	0.40	0.28		0.60		0.80		0.40		1.10	0.006	0.40	0.28	0.60	0.70	0.40	1.40	0.011
PT (sec)	15.20	14.00		16.40		14.50		13.55		16.05	0.444	14.80	13.65	16.25	16.50	15.40	16.80	0.002
INR	1.22	1.10		1.35		1.17		1.05		1.33	0.407	1.21	1.07	1.33	1.35	1.28	1.38	0.008

*ALU ratio* arthrobacter luteus ratio, *x* Median, *WBCs* white blood cells, *Hb* hemoglobin, *AST* aspartate aminotransferase, *ALT* alanine aminotransferase, *AFP* alpha-fetoprotein, *PT* platelets, *INR* International normalized ratio

### Correlation between patients’ demographic and clinical features and different TM6SF2 rs58542926 genotypes involved in genomic and circulating DNA

The association between different TM6SF2 rs58542926 genotypes with the clinical and demographic characteristics of 147 HCC patients is displayed in Table 3. Hepatocellular carcinoma stage progression and the CC genotype in genomic DNA are significantly correlated, according to the BCLC staging system. Regarding alcohol consumption, the circulating DNA of alcoholic consumers is dominated by the CT genotype, whereas the genomic DNA of non-alcoholic consumers shows a large amount of the CC genotype. The CT genotype in circulating DNA is significantly correlated with smoking and HCV-positive PCR, as is the case in individuals with multiple focal lesions in the liver.

**Table 3:** Demographic and Clinical features of patients at HCC diagnosis concerning different TM6SF2 rs58542926 genotypes in genomic and circulating DNA

	Genomic			Circulating		
	CC (*n* = 135)	CT (*n* = 12)	*P* value	CC (*n* = 132)	CT (*n* = 15)	*P* value
BMI Median (1 st quartile-3rd quartile)	25.30 (23.50- 27.40)	25.35 (23.55- 27.15)	0.975	25.35 (23.0–27.40)	24.30 (24.10–26.40)	0.751
Child score point Median (1 st quartile-3rd quartile)	6.00 (5.00–7.0)	5.50 (5.00–6.50)	0.658	6.00 (6.00–7.00)	6.00 (5.00–6.00)	0.782
Characteristic (Count, Row N%)						
HCC Stage						
Early	66 (91.7%)	6 (8.3%)	0.941	63 (87.5%)	9 (12.5%)	0.368
Late	69 (92.0%)	6 (8%)		69 (92.0%)	6 (8.0%)	
BCLC						
0	21 (77.8%)	6 (22.2%)	0.003	24 (88.9%)	3 (11.1%)	0.625
A	45 (100.0%)	0 (0%)		39 (86.7%)	6 (13.3%)	
C	69 (92.0%)	6 (8.00%)		69 (92.0%)	6 (8.0%)	
Child score class						
A	96 (91.4%)	9 (8.6%)	1	93 (88.6%)	12 (11.4%)	0.556
B	39 (92.9%)	3 (7.1%)		39 (92.9%)	3 (7.1%)	
Gender						
Female	27 (90%)	3 (10%)	0.711	27 (90.0%)	3 (10%)	1
Male	108 (92.3%)	9 (7.7%)		105 (89.7%)	12 (10.3%)	
DM						
Yes	57 (95.0%)	3 (5.0%)	0.361	57 (95.0%)	3 (5.0%)	0.083
No	78 (89.7%)	9 (10.3%)		75 (86.2%)	12 (13.8%)	
Smoking						
Yes	72 (88.9%)	9 (11.1%)	0.148	69 (85.2%)	12 (14.8%)	0.041
No	63 (95.5%)	3 (4.5%)		63 (95.5%)	3 (4.5%)	
Alcoholic intake						
Yes	3 (50.0%)	3 (50.0%)	0.007	0 (0.0%)	6 (100%)	< 0.001
No	132 (93.6%)	9 (6.4%)		132 (93.6%)	9 (6.4%)	
HCV PCR						
Positive	57 (90.5%)	6 (9.1%)	0.602	51 (81.0%)	12 (19.0%)	0.004
Negative	78 (92.9%)	6 (7.1%)		81 (96.4%)	3 (3.6%)	
DAAS						
Yes	78 (89.7%)	9 (10.3%)	0.361	81 (93.1%)	6 (6.9%)	0.111
No	57 (95.0%)	3 (5%)		51 (85.0%)	9 (15.0%)	
Family history of HCC						
Yes	24 (100%)	0 (0%)	0.216	21 (87.5%)	3 (12.5%)	0.713
No	111 (90.2%)	12 (9.8%)		111 (90.2%)	12 (9.8%)	
Performance Status						
0	84 (90.3%)	9 (9.7)	0.644	81 (87.1%)	12 (12.9%)	0.464
> 1	48 (94.1%)	3 (5.9%)		48 (94.1%)	3 (5.9%)	
1	3 (100%)	0 (0%)		3 (100.0%)	0 (0.0%)	
Liver size						
Shrunken	9 (100%)	0 (0.0%)	0.149	9 (100.0%)	0 (0.0%)	0.606
Enlarged	66 (95.7%)	3 (4.3%)		63 (91.3%)	6 (8.7%)	
Average	60 (87.0%)	9 (13.0%)		60(87.0%)	9 (13.0%)	
No. of focal lesions						
Single	78 (89.7%)	9 (10.3%)	0.118	81 (93.1%)	6 (6.9%)	< 0.001
Two	33 (100.0%)	0 (0.0%)		33 (100.0%)	0(0%)	
Multiple	24(88.9%)	3 (11.1%)		18 (66.7%)	9 (33.3%)	
Portal vein						
Thrombosed	24 (100.0%)	0 (0.0%)	0.216	21 (87.5%)	3 (12.5%)	0.713
Patent	111 (90.2%)	12 (9.8%)		111 (90.2%)	12 (9.8%)	
Malignant lymph nodes						
Yes	21 (100.0%)	0.0 (0%)	0216	21 (100%)	0 (0%)	0.129
No	114 (90.5%)	12 (9.5%)		111 (88.1%)	15 (11.9%)	
Ascites						
Yes	30 (90.9%)	3 (9.1%)	0.732	30 (90.9%)	3 (9.1%)	1
No	105 (92.1%)	9(7.9%)		102 (89.5%)	12 (10.5%)	
Previous ablation						
Yes	63 (91.3%)	6 (8.7%)	0.825	63 (91.3%)	6 (8.7%)	0.570
No	72 (92.3)	6 (7.8%)		69 (88.5%)	9 (11.5%)	

*DM* Diabetes Miletus, *DAAS* Direct Acting Anti-hepatitis C Virus Drugs

### Multivariate analysis of demographic and clinical factors associated with harboring CT genotype in HCC patients

Risk factors for HCC were assessed in the population being studied, as indicated in Table 4. A univariate analysis showed that smoking, ALT, INR, prothrombin time, numerous focal lesions, and positive-HCV PCR were statistically significant risk factors. However, a number of localized lesions and a positive HCV PCR were the only indicators that could independently predict the CT genotype in circulating DNA.

**Table 4:** Multivariate logistic regression to detect independent predictors of CT genotype in circulating DNA

	*P* value	OR	95% C.I	
			Lower	Upper
CT Circulating DNA				
ALT	0.821	0.998	0.981	1.015
Prothrombin time	0.625	1.237	0.528	2.896
INR	0.609	0.202	0.000	92.576
Smoking (yes)	0.158	3.378	0.622	18.339
HCV PCR (+ ve)	0.040	4.886	1.079	22.133
No. of focal lesions (multiple)	0.042	5.069	1.057	24.311

### Analyzing samples (n = 15) whose TM6SF2 rs58542926 genotypes switched from CC to CT or from CT to CC

The TM6SF2 genotypes in 15 samples were found to have shifted from CC to CT or from CT to CC (genomic to circulating free DNA or vice versa), This change should have been considered. In contrast to a notable increase in AST and ALT activity, direct and total bilirubin concentration, and child score point in the same samples, we observed a substantial decrease in hemoglobin concentration and albumin in patient serum whose genotypes were shifted (15 samples). Genomic or circulating genotypes were significantly altered in samples with HCV-positive PCR results. On the other hand, genotype of patients treated with DAAS remains mostly unaltered in their genomic and circulating samples. Liver enlargement and maintaining the genotype unchanged were found to be significantly correlated. The presence of liver focal lesions strongly reduces the chance of maintaining the unchanged genotype in both genomic and circulating DNA (Table 5).

**Table 5:** Relation between patients’ characteristics and TM6SF2 rs58542926 genotyping change in 15 samples

	changed genotypes (n = 15)	same genotypes (n = 132)	*P*-value
Characteristic (Mean, ± Standard deviation)			
ALU ratio	1.26 ± 0.15	1.33 ± 0.06	0.340
WBCs (x* 10^3^)	7.44 ± 3.76	5.35 ± 2.02	0.074
Hb (mg/dL)	11.90 ±	12.81 ± 1.86	0.009
Platelets (x/mm^3^)	140.40 ± 70.25	140.89 ± 68.39	0.863
AST (U/L)	86.60 ± 39.35	52.41 ± 28.65	0.001
ALT (U/L)	57.20 ± 7.48	46.80 ± 33.29	0.001
Creatinine (mg/dL)	1.14 ± 0.41	1.00 ± 0.28	0.088
AFP (μg/L)	472.10 ± 3.61	100.92 ±	0.508
Albumin (g/dL)	3.00 ± 0.46	3.61 ± 0.62	< 0.001
Total bilirubin (mg/dL)	1.66 ± 0.51	1.10 ± 0.47	< 0.001
Direct bilirubin (mg/dL)	1.00 ± 0.29	0.44 ± 0.29	< 0.001
PT (sec)	15.40 ± 1.78	15.30 ± 2.13	0.665
INR	1.22 ± 0.15	1.26 ± 0.27	0.863
BMI	25.18 ± 2.07	25.65 ± 3.55	0.818
Child score point	6.60 ± 0.83	5.86 ± 1.08	0.002
Characteristic (Count, Row N%,)			
HCC Stage			
Early	9 (60.0%)	63 (47.7%)	0.368
Late	6 (40.0%)	69 (52.3%)	
BCLC			
0	3 (20.0%)		0.625
A	6 (40.0%)		
C	6 (40.0%)		
Child score class			
A	)960.0%(	96)72.7%(	0.366
B	)640.0%(	36) 27.3%(	
Gender			
Female	6 (40.0%)	24 (18.2%)	0.083
Male	9 (60.0%)	108(81.8%)	
DM			
Yes	6 (40.0%)	54 (40.9%)	0.946
No	9 (60.0%)	78 (59.1%)	
Smoking			
Yes	9 (60.0%)	72 (54.5%)	0.687
No	6 (40.0%)	60 (45.5%)	
Alcoholic intake			
Yes	3 (20.0%)	3 (2.3%)	0.014
No	12(80.0%)	129(97.7%)	
HCV PCR			
Positive	)1280.0%(	51) 38.6%	0.002
Negative	)320.0%(	81) 61.4%	
DAAS			
Yes	)320.0%(	84) 63.6%(	0.001
No	)1280.0%(	48) 36.4%(	
Family history of HCC			
Yes	)320.0%(	21)15.9%(	0.713
No	)1280.0%(	111) 84.1%(	
Performance status			
0	)960.0%(	84) 63.6%(	0.839
> 1	)640.0%(	45) 34.1%(	
< 1	)00.0%(	3) 2.3%(	
Liver size			
Shrunken	)00%(	9)6.8%(	0.028
Enlarged	)320.0%(	66) 50.0%(	
Average	)1280.0%(	57) 43.2%(	
No. of focal lesions			
Single	)960.0%(	78)59.1%(	0.009
Two	)00%(	33) 25.0%(	
Multiple	)640.0%(	21)15.9%(	
Portal vein			
Thrombosed	)320.0%(	21)15.9%(	0.713
Patent	)1280.0%(	111)84.1%(	
Malignant lymph nodes			
Yes	)00%(	21)15.9%(	0.129
No	)15,100%(	111)84.1%(	
Ascites			
Yes	)640.0%(	27)20.5%(	0.104
No	)960.0%(	105)79.5%(	
Previous ablation			
Yes	)640.0%(	63)47.7%(	0.570
No	)960.0%(	69)52.3%(	

*x* Median, *WBCs* white blood cells, *Hb* hemoglobin, *AST* aspartate aminotransferase, *ALT* alanine aminotransferase, *AFP* alpha-fetoprotein, *PT* platelets, *INR* International normalized ratio, *DM* Diabetes Miletus, *DAAS* Direct Acting Anti-Hepatitis C Virus Drugs

### Kaplan Meier curve for association of TM6SF2 polymorphism in genomic and circulating DNA with survival

#### Analyzing different genotypes in genomic samples

**Fig. 1:**
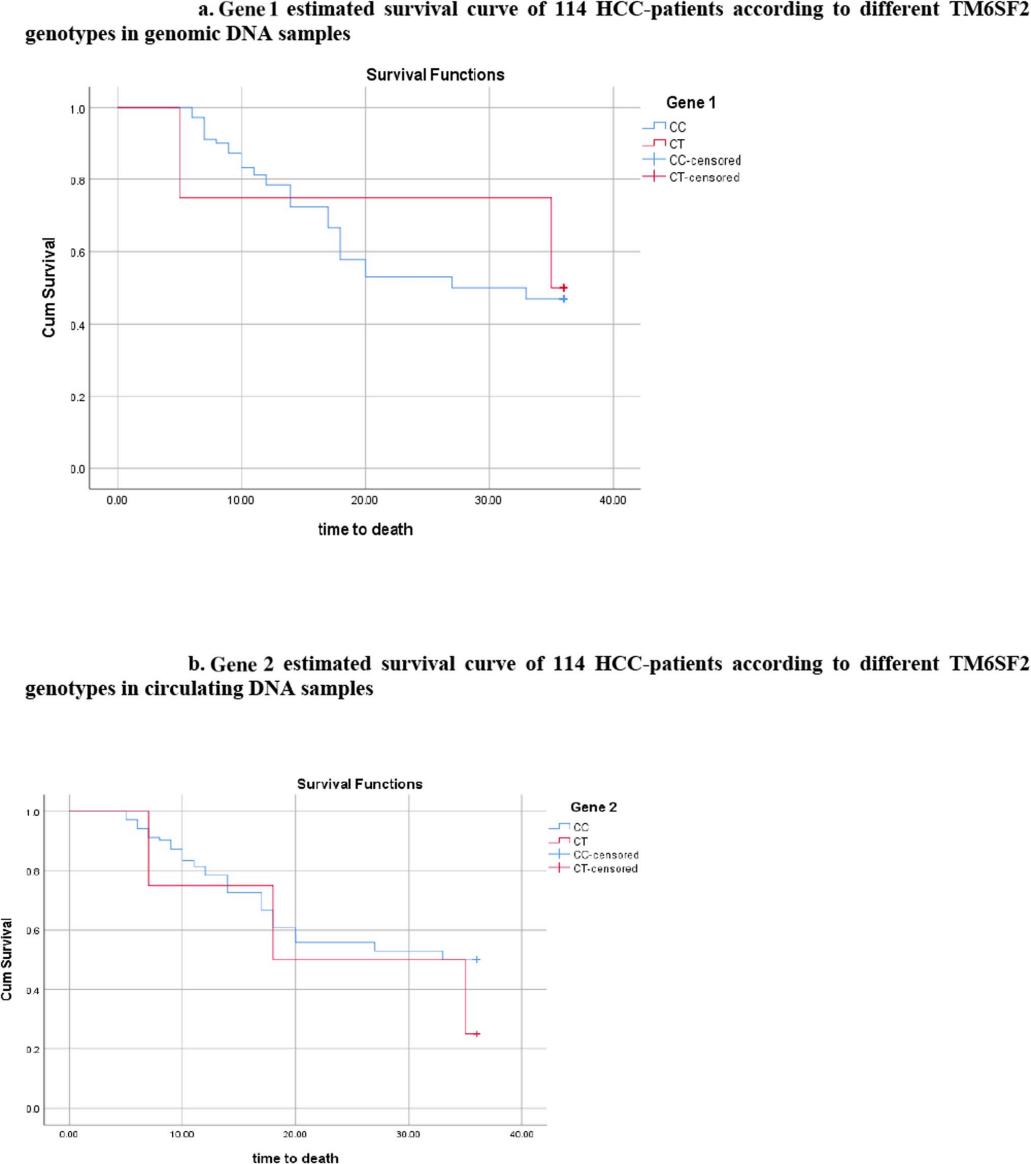
**a** Kaplan–Meier estimated survival curve of 114 HCC-patients according to different TM6SF2 genotypes in genomic DNA samples. **b** Kaplan–Meier estimated survival curve of 114 HCC-patients according to different TM6SF2 genotypes in circulating DNA samples

By the end of the study, 54 patients with CC genotype were dead compared to 48 patients who remained alive, while patients with CT genotype were equally distributed between dead or alive (6 patients in each group) and 33 patients could not be followed. Fig. 1a. shows that there was no significant correlation between the survival rate and the various genotypes (P 0.802).

#### Analyzing different genotypes in circulating samples

In contrast to nine individuals with the CT genotype who died and three who survived, fifty-one patients with the CC genotype died and the same number survived. Figure 1b shows that there was no significant correlation between the survival rate and the various genotypes (P 0.216).

## Discussion

Transmembrane 6 Superfamily Member 2 (TM6SF2) gene encodes an endoplasmic membrane protein that is mainly expressed in the liver as well as the gut and both kidneys. The gene is located on chromosome 19. The produced protein is involved in hepatic lipid metabolism, triglyceride secretion and hepatic intracellular lipid droplet concentration [10, 20–22]. Dysregulation of underlying genetic factors can result in hepatic lipid accumulation, steatosis and promotion of HCC development [23]. TM6SF2 variation happens by a C-to-T substitution at the nucleotide 499 that encodes a glutamate-to-lysine change at codon 167. The produced misfolded protein will accelerate protein degradation and subsequently reduce protein levels [24]. The variant gene enhances the expression of several inflammatory cytokines such as IL-2 and IL-6 and accelerates HCC progression [25]. Focusing on studying genotyping and genetic polymorphisms can possibly lead to fruitful and precise HCC risk stratification and screening with a productive optimization of patient care [26].

Youssef et al. study included 264 patients (120 HCC patients and 144 HCV patients), TT genotypes predominated with HCC groups and the T allele significantly contributed to the different stages of HCC [10]. Similarly, another study demonstrated a significant correlation between HCC and both TT genotype and T allele. CT genotype was more prevalent in cirrhotic patients [21]. In another study that involved 40 HCC patients, 40 CLD patients and another 40 healthy controls, frequencies of T allele were higher in HCC patients than in the other two groups (OR 5.44). A higher risk of developing HCC was found in CLD patients with the CT genotypes (OR 4.67) and the TT genotypes (OR 9.33) [22]. In our study, we focused on the genetic variation in the HCC patients who developed malignancy on a background of HCV-related chronic liver disease. Certainly, we looked for the genotype distribution of the TM6SF2 in HCC patients. The CC genotype was predominant. Genomic distribution of CC and CT were 135 and 12 patients, respectively. Circulating CC and CT were 132 and 15 patients, respectively.

As we looked for correlations that could be present between the TM6SF2 variation and the patients characteristics as well as the tumor characteristics, we found that patients with the CT genotype had significantly higher serum direct bilirubin, total leucocyte count, and hemoglobin than the CC genotype patients. On the other hand, circulating samples in patients with the CT genotype had significantly higher serum direct bilirubin, ALT, and prothrombin time than CC genotype patients, as well as a significantly higher percentage of smoking, alcohol intake, and HCV PCR seropositivity. The CT genotype significantly correlated with tumor multi-centricity. This gives the impression that the CT genotype is related to the different predisposing carcinogenic factors as well as the progressive stage of HCC. Multivariate analysis declared the statistical significance of HCV PCR seropositivity and the multi-centricity as factors related to CT genotype. Even more important, the type of change from CT to CC genotype significantly correlated with HCV PCR seronegativity, the intake of DAAs, the presence of single focal lesions and the better levels of hemoglobin, platelet count, ALT enzyme, prothrombin and INR. This ensures the same conclusion that CT genotype carries the worse outcome and impact on liver and HCC while the shift to CC genotype improves the status of the patient and the tumor. To our knowledge, our paper is the first to prove the impact of shift of CT to CC genotype. Revising the literature, we just found a study where creatinine level was significantly lower in the TT genotype of cancer patients [21].

The TM6SF2 variant is a key player in the whole spectrum of NAFLD and ALD [27, 28]. In Paternostro et al. study that included 703 patients, TM6SF2 was associated with advanced fibrosis. Incorporation of the gene to different genetic markers to predict advanced fibrosis led to higher AUC (0.786) [29]. In the opposite, another study included 515 NAFLD patients and concluded that the TM6SF2 genotype correlated with steatosis while no correlation occurred with fibrosis [30]. In a systemic review and meta-analysis study that included 4325 HCV patients, the TM6SF2 variant correlated with steatosis, fibrosis and cirrhosis while no correlation occurred with severe inflammation. HCV PCR levels did not differ in patients with variants and non-variants for TM6SF2 [31]. In Oliveira et al. study, other factors proved more influential than the TM6SF2 polymorphism in relation to hepatic steatosis or fibrosis. Univariate analysis found various factors such as old age, the presence of diabetes or insulin resistance, arterial hypertension, dyslipidemia, metabolic syndrome and liver enzymes (ALT, AST, Alkaline phosphatase and GGT) [32]. Finally, a large meta-analysis study that gathered data from 123.800 persons (across 44 studies) found that the T allele increased the risk of NAFLD in children and adults. This involved steatosis progression and fibrosis progression [27]. In the era of DAAs with high SVR rates, the identification of genetic polymorphism and other metabolic characteristics of HCV patients might be of utility for risk stratification after treatment. As fibrosis correlated with TM6SF2 in association with factors of metabolic syndrome, independent of the presence of HCV, attention after HCV eradication is important. Moreover, the combination of biomarkers for predicting HCC risk in conjunction with lifestyle change techniques may ultimately offer a reliable and economical method for HCC prevention in at-risk individuals with chronic liver disease. Consequently, reducing the burden of this extremely deadly illness requires incorporating lifestyle messaging into patient counseling for those who are at risk for HCC.

Our study had several points of strength. We studied the influence of change, either CC-to-CT or CT-to-CC, on patients and HCC characteristics. We did HCC staging and tried to correlate it with the results of genotyping. We focused on HCV as cause of underlying liver disease to avoid bias by other factors such as HBV. We did a survival analysis to ensure if these changes could have further implementation on HCC prognosis of studied patients. However, we had certain limitations. We lacked the data of different metabolic factors such as distribution of body fat and dyslipidemia. Only Diabetes was assessed in our patients. Still, our study is a single center study and further validation in a multi-center would be beneficial. Future research should go at examining other HCC-related genetic variants to see how they interact to affect the course of the disease.

## Conclusion

In conclusion, our study provides evidence for the association between the TM6SF2 rs58542926 gene polymorphism and the development of HCC in patients with HCV infection. The CC genotype was found to be the most common in our HCC patients, and the CT genotype was associated with higher serum direct bilirubin, total leucocyte count, and hemoglobin than the CC genotype patients. Our findings suggest that lifestyle factors such as smoking and alcohol intake may contribute to the development of HCC in patients with CT genotype. In addition, the shift from CT to CC genotype correlated with better laboratory results, HCV PCR negativity and better tumor characteristics. These results may help to inform future research and clinical practice in the diagnosis and treatment of HCC in patients with HCV infection.

## Supplementary Information

Additional file 1: Figure 1. a. Kaplan-Meier estimated survival curve of 114 HCC-patients according to different TM6SF2 genotypes in genomic DNA samples. Figure 1. b. Kaplan-Meier estimated survival curve of 114 HCC-patients according to different TM6SF2 genotypes in circulating DNA samples

## Data Availability

All data generated or analyzed during this study are included in this published article [and its Additional file 1].
