# Supplementary material for: Impact of differential detection of TM6SF2 rs58542926 mutation in circulating tumor DNA versus peripheral blood cells on hepatocellular carcinoma patients
Source: Discov Oncol. 2025 Jun 12;16:1071. doi: 10.1007/s12672-025-02812-9 (PMC12162427; doi:10.1007/s12672-025-02812-9)

**Figure 1. a. Kaplan-Meier estimated survival curve of 114 HCC-patients according to different TM6SF2 genotypes in genomic DNA samples**


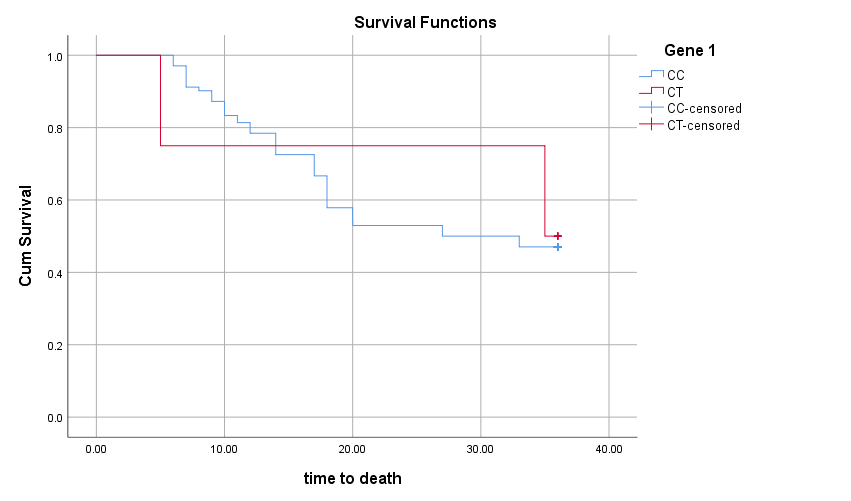


**Figure 1. b. Kaplan-Meier estimated survival curve of 114 HCC-patients according to different TM6SF2 genotypes in circulating DNA samples**


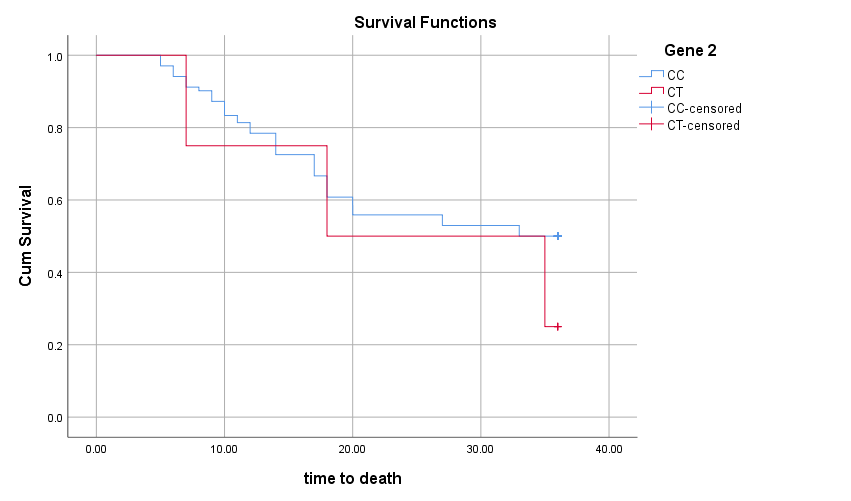

Supplement: Supplementary file 1 — Additional file 1: Figure 1. a. Kaplan-Meier estimated survival curve of 114 HCC-patients according to different TM6SF2 genotypes in genomic DNA samples. Figure 1. b. Kaplan-Meier estimated survival curve of 114 HCC-patients according to different TM6SF2 genotypes in circulating DNA samples [file 12672_2025_2812_MOESM1_ESM.docx]
